# The impact of presidents’ transformational leadership on faculty occupational well-being—parallel mediating roles of teaching efficacy and job crafting

**DOI:** 10.3389/fpsyg.2025.1468563

**Published:** 2025-06-06

**Authors:** TingYu Sun, Yang Luo, Mankeun Yoon

**Affiliations:** ^1^Department of Labor Relations, Shandong Management University, Jinan, China; ^2^Department of Education, The Catholic University of Korea, Bucheon-si, Republic of Korea

**Keywords:** occupational well-being, transformational leadership, job crafting, teaching efficacy, higher education

## Abstract

**Introduction:**

Occupational well-being (OW) has gained increasing attention in recent years; however, limited research has focused on university and college faculty—despite their high levels of burnout and job dissatisfaction. This study examines the impact of university presidents’ transformational leadership (TL) on faculty OW, and explores the mediating roles of job crafting and teaching efficacy in this relationship.

**Methods:**

A total of 555 faculty members from various higher education institutions in Shandong Province, China, voluntarily participated in an online survey. CB-SEM was applied to examine direct and indirect relationships among the variables, with bootstrapping techniques employed to test mediation effects.

**Results:**

The results revealed that: (1) presidents’ TL significantly and positively predicted faculty OW; (2) job crafting partially mediated the relationship between TL and OW; and (3) teaching efficacy also partially mediated this relationship.

**Discussion:**

These findings suggest that transformational leadership by university presidents not only directly enhances faculty well-being but also exerts indirect effects through job crafting and teaching efficacy. This study provides valuable insights for higher education administrators seeking to promote faculty well-being through effective leadership strategies.

## 1 Introduction

Faculty members are central to the development of higher education and, as a result, are often subject to significant societal attention. Their occupational well-being (OW) is a key indicator of both their psychological health and the overall quality of their professional experience (Capone and Petrillo, 2020). Despite their importance, the current state of faculty well-being has shown a declining trend. Faculty members frequently face excessive workloads, role ambiguity, limited autonomy, and a diminished sense of job satisfaction (Meeks et al., 2023; Smith et al., 2022). These conditions have led to growing instances of occupational stress, anxiety, depression, and burnout (Meeks et al., 2023). As such, addressing faculty well-being is essential to ensuring their long-term stability and sustaining the effectiveness of the higher education system.

Research on teacher well-being typically focuses on three levels: individual, societal, and organizational. At the individual level, factors such as organizational commitment (Wessels and Wood, 2019), self-efficacy (Shoji et al., 2016), and motivation (Collie and Martin, 2017) are key. Societal-level factors include social support (Hur et al., 2016) and interpersonal relationships (Milatz et al., 2015). At the organizational level, leadership style (Ghamrawi et al., 2023) and perceived support (Soykan et al., 2019) have been shown to play influential roles.

This study focuses on the impact of organizational factors on teacher well-being. The organizational structure and leadership identity of Chinese higher education institutions exhibit distinctive characteristics. In contrast to many Western systems, China employs a presidential accountability model under the leadership of the Communist Party of China (CPC). University presidents act as legal representatives and chief administrators, overseeing academic, teaching, and administrative functions (Liu et al., 2019). They typically possess broad authority, including influence over personnel decisions, departmental restructuring, performance evaluations, and job roles, often maintaining close interactions with faculty (Yu et al., 2024). This strong administrative structure suggests that university leadership may have a particularly significant impact on faculty well-being. Prior research indicates that leadership styles that respect and support professional development contribute positively to well-being (Cann et al., 2021).

Transformational leadership (TL) is one such style. It emphasizes vision, inspiration, individualized support, and the development of follower potential (Bass and Avolio, 1994). TL has been associated with increased creativity (Watts et al., 2020), reduced burnout (Heidmets and Liik, 2014), and enhanced attitudes, beliefs, and behaviors in educational contexts (Runhaar et al., 2010). TL also contributes to greater job satisfaction and a sense of accomplishment by fostering intrinsic motivation and value alignment (Luyten and Bazo, 2019). Accordingly, we hypothesize a significant association between presidents’ transformational leadership and faculty OW in Chinese higher education. Faculty face multifaceted demands beyond teaching, including research, administration, funding acquisition, and institutional service. These pressures can lead to burnout and diminished well-being (Lashuel, 2020). Job crafting (JC)—defined as proactive adjustments to job tasks, relationships, or perceptions—offers a coping strategy that helps improve work engagement and well-being (Stempel and Siestrup, 2022; Tims et al., 2013; van Wingerden et al., 2017). Additionally, teaching efficacy (TE), or faculty members’ belief in their ability to foster student learning, plays a vital role in motivation, teaching performance, and well-being (Wang et al., 2015). Both job crafting and teaching efficacy are thus considered critical in promoting faculty OW.

The Job Demands-Resources (JD-R) model provides a theoretical framework for understanding how job demands and resources affect employee outcomes via two pathways: the impairment process (demands leading to strain) and the motivational process (resources enhancing engagement). University leadership influences faculty’s access to resources and exposure to demands (Hetland et al., 2018), while job crafting enables employees to reshape their work environment (Rudolph et al., 2017). Teaching efficacy, as a personal resource, also interacts with work conditions to influence well-being (Bakker et al., 2023). Thus, transformational leadership, job crafting, and teaching efficacy may affect occupational well-being through JD-R mechanisms.

Although prior studies have linked TL to well-being, many emphasize mental health (Hannah et al., 2020; Irshad et al., 2021) or subjective well-being (Heidmets and Liik, 2014; Soni, 2022), with relatively few focusing specifically on occupational well-being. Furthermore, research on these mechanisms in the context of Chinese higher education remains limited. Most studies focus on primary and secondary school teachers, overlooking faculty in universities. Given the distinctive administrative structure of Chinese higher education and the pivotal role faculty play in student success and institutional outcomes, it is essential to explore faculty well-being in this context. Doing so also contributes to the cultural and contextual expansion of the JD-R framework. This study investigates whether transformational leadership by university presidents predicts faculty occupational well-being in Chinese higher education. It further explores the mediating roles of job crafting and teaching efficacy in this relationship, using JD-R theory as the guiding framework.

## 2 Literature review and hypotheses

### 2.1 Job demands-resources theory

The JD-R theory is a job design theory proposed by Demerouti et al. (2001), which integrates various perspectives on job stress and motivation and explains how specific physical, social, or psychological characteristics of a job and an organization can affect employee well-being, and consequently, employee health, behavior, and performance (Bakker and Demerouti, 2017). The first proposition of the JD-R theory is that while the characteristics of each organization entity may vary, they can all be conceptualized using two overarching categories: job demands (such as workload, conflicting responsibilities, and emotional requirements) and job resources (such as social support, participation in decision-making, and leadership commitment). The second proposition of the JD-R is that job demands and job resources trigger an “impairment process” and a “motivational process.” The impairment process refers to job demands deplete employees’ physical, emotional, and cognitive resources, leading to fatigue and health problems that diminish employees’ well-being. The motivational process refers to job resources that satisfy fundamental psychological requirements, promote employee engagement in the job, and lead to increased creativity and performance, thus enhancing employees’ well-being. The dual-path hypothesis of the JD-R model posits that the enhancement of job resources and the minimization of job demands can lead to an improvement in employees’ OW (Bakker et al., 2023).

### 2.2 Transformational leadership and occupational well-being

As defined by Bass (1990), TL refers to the exceptional leadership performance that occurs when leaders expand and elevate the concerns of their followers, enlighten them about and encourage their commitment to the team’s purpose and mission, and inspire their followers to prioritize the team’s welfare over their own self-interests. Bass (1995) classified TL into four fundamental dimensions: idealized influence, inspirational motivation, intellectual stimulation and individualized consideration. Influenced by Confucian thought, China places particular emphasis on the element of moral character across various domains, with management being no exception. Chinese scholars have found that within the Chinese cultural context, moral elements are an integral part of the structure of transformational leadership (Li and Shi, 2005). Therefore, this study defines presidents’ TL as behaviors exhibited by presidents who utilize idealized influence, ethical conduct, and individualized consideration to cultivate a shared vision with faculty, raise faculty awareness of the significance of their tasks, and stimulate higher-order needs and intrinsic motivation in faculty to continually transcend themselves.

OW encompasses positive emotions and positive appraisals of emotional, motivational, behavioral, and cognitive factors within the workplace (Van Horn et al., 2004). Carine and Pablo (2020) defined teachers’ OW as a cognitive, subjective, health and social state of being that is related to their vocation of teaching. Some scholars define teachers’ OW as a psychological feeling and mental state of sustained happiness in their professional life in which their needs are met, their potential is fulfilled, and their self-worth is realized. In this study, faculty OW is defined as a positive state characterized by the fulfillment of personal needs, a sense of self-worth, comfort within the organizational environment, and the attainment of both physical and mental health.

Some studies have demonstrated that TL inspires followers to develop positivity and optimism through ethics and idealized influence (Lee et al., 2024), and improves followers’ need satisfaction through vision building and individualized care (Kieres, 2013). TL helps facilitates the resolution of problems and challenges, as well as the enhancement of professional competence (Thomas and Aurora, 2023). As previously mentioned, positivity and optimism (Brouhier et al., 2023), need satisfaction (Capone and Petrillo, 2020), and professional competence (Harrison et al., 2023) are all components of OW. Moreover, leaders can serve as pivotal job resources, such as individualized consideration and intellectual stimulation (Breevaart and Bakker, 2018), which can markedly enhance employees’ OW. Additionally, empirical studies have corroborated the aforementioned theories. For instance, TL has been demonstrated to enhance OW by modifying employees’ perceptions of job characteristics and job motivation (Fernet et al., 2015). Many other studies have incorporated additional moderating or mediating variables to investigate the relationship between TL and OW. These include efficacy (Liu et al., 2019; Nielsen and Munir, 2009), degree of demand satisfaction (Stenling and Tafvelin, 2014) and personality traits (Zadok et al., 2024) etc. In consideration of the aforementioned evidence, this study posits that presidents’ TL may serve as a significant determinant in promoting faculty OW. Consequently, the following hypothesis is proposed:

*H1*: Presidents' Transformational Leadership (TL) positively predict faculty Occupational Well-being (OW).

### 2.3 The mediating role of job crafting

The concept of Job Crafting (JC) also comes from the exploration of job design theory. Wrzesniewski and Dutton (2001) initially proposed the role theory perspective of job crafting, which posits that employees utilize their own abilities and strengths, take the initiative to make changes and redesign their jobs in order to align the job more compatible with their own abilities and strengths. From the perspective of job resources theory, Tims et al. (2013) proposed that by continually crafting their job demands to align with their resources and reconceptualizing job content, employees can enhance the compatibility of their roles with their abilities and expectations. They categorize job crafting into three dimensions: increasing job resources, increasing challenging job demands, and reducing hindrance job demands. This study uses the definition and structure proposed by Tims et al. (2013).

The sixth proposition of JD-R theory posits that employees engage in proactive optimization of their job demands and resources through job crafting (Bakker et al., 2023). Job crafting enhances person-job fit, augments job engagement and elevates performance by optimizing job characteristics (Moreira et al., 2022). As posited by Tims et al. (2013), job crafting can increase job resources, increase challenging job demands, and decrease hindrance job demands, where job resources and challenging job demands are thought to have a beneficial influence, and hindrance job demands are thought to have a detrimental influence. Therefore, job crafting has the potential to stimulate the motivational process in JD-R by increasing job resources and increasing challenging job demands, while inhibiting the impairment process in JD-R by reducing hindrance demands. Job design is one of the most important ways to improve employee performance and well-being (Parker et al., 2017), faculty OW can be bolstered through job crafting, which will bring them better job design, more meaningful work, reduced burnout, heightened job dedication, and elevated performance. In light of the aforementioned evidence, we posit that job crafting may positively affect faculty OW.

The characteristics of TL, including the provision of support for employee development and the facilitation of the expectations exceeding and obstacles overcoming, can contribute to the promotion of proactive behaviors (Moreno-Casado et al., 2022). Job crafting is one such proactive bottom-up change behavior of employees. Leaders who are motivated and attentive to the individual needs of their employees facilitate a culture of responsibility, autonomy, and motivation. They encourage employees to take ownership of their work and optimize their work environment (Breevaart and Bakker, 2018). TL encourages employees to adopt new operational models, motivates them to identify novel approaches to their work, and anticipates enhanced performance (Wang et al., 2017). Moreover, numerous scholars have corroborated the positive correlation between TL and job crafting through empirical investigation (Naeem et al., 2020; Wojtczuk-Turek, 2022). In light of the aforementioned evidence, this study posits that presidents’ TL may serve as a potential antecedent variable of job crafting.

Overall, TL facilitate employee engagement in challenging roles and promote a willingness to challenge established paradigms of thinking and operating (Den Hartog and Belschak, 2012), promoting job crafting among faculty. This practice enables them to align their work more closely with their capabilities and preferences, thereby modifying the content, methods, and value of their job, and consequently reducing hindrance job demands (Rudolph et al., 2017). In summary, presidents’ TL can enhance faculty OW by facilitating job crafting, stimulating the motivational process in JD-R, and inhibiting the impairment process in JD-R. In light of the aforementioned evidence, the following hypothesis is proposed:

*H2*: Job Crafting mediates the relationship between Presidents' Transformational Leadership (TL) and faculty's Occupational Well-being (OW).

### 2.4 The mediating role of teaching efficacy

According to Bandura’s social cognitive theory and self-efficacy theory, self-efficacy refers to an individual’s subjective assessment, judgment, and prediction of whether or not he or she can successfully complete a task or accomplishment (Bandura, 1977). This subjective judgment (self-efficacy) not only influences one’s confidence and beliefs about success, but also determines one’s motivation, actions, and physical and psychological responses when faced with negative situations (Bandura, 2001). Teaching Efficacy (TE) is defined as the judgment and feelings of teaching staff regarding their ability to teach and to positively influence student learning outcomes and behaviors (Nail et al., 2015). Yu et al. (1995) defined teaching efficacy as teaching staff subjective judgment of their ability to accomplish teaching tasks and achieve teaching goals in the teaching process based on China’s national conditions. They divided teaching efficacy into two dimensions: Personal Teaching Efficacy and General Teaching Efficacy. This study uses the definition and structure proposed by Yu et al. (1995).

The fourth proposition in the JD-R theory states that personal resources such as self-efficacy have a reciprocal relationship with job resources, which means that employees with more personal resources are also more likely to have more job resources (Bakker et al., 2023). Therefore, increased teaching efficacy helps faculty optimize their job resources and stimulate the motivational process of JD-R, resulting in more OW. Furthermore, the advent of digital modern education has introduced a new set of challenges for faculty, who are now confronted with the complexities of technology and the potential for technological insecurity (Li and Wang, 2021). These factors affect the performance and act as stressors on the well-being of faculty (Salo et al., 2019). The extant literature indicates that teachers’ self-efficacy is a significant predictor of their well-being, job satisfaction, and intention to leave the profession (Wang et al., 2015). The relationship has also been examined empirically, indicating that components of teacher self-efficacy related to teaching efficacy are positively correlated with various dimensions of their well-being (Reppa et al., 2023). A teacher who perceives that they possess the capacity to engage students in learning will experience higher job satisfaction and lower burnout (Malinen and Savolainen, 2016). In consideration of the aforementioned evidence, this study posits that teaching efficacy represents an intrinsic factor in OW.

A study has demonstrated that presidents’ TL in teaching and learning has a significant positive effect on all three sub-dimensions of teaching efficacy: classroom management efficacy, teaching strategy efficacy, and student engagement efficacy (Bellibas and Liu, 2017). The level of teaching efficacy was higher when all six behaviors of TL were present (Lilla, 2013). Furthermore, the higher the TL, the higher the level of both general teaching efficacy and personal teaching efficacy (Choi and Sung, 2011). In consideration of the aforementioned evidence, this study posits that TL may serve as a potential antecedent variable of teaching efficacy.

Overall, TL, as a forward-thinking leadership style, has been shown to encourage innovation, personal growth, and social interaction among employees, thereby fostering intrinsic motivation, enhancing confidence, and promoting creativity. This approach has been found to contribute to an enhancement in teaching efficacy among faculty (Al Harbi et al., 2019). As a core personal resource of faculty in teaching work, teaching efficacy has a mutually beneficial relationship with job resources, and is one of the important “positive factors” in the work, which helps to stimulate the motivational process of JD-R (Bakker et al., 2023). In summary, presidents’ TL can contribute to enhance faculty OW by improving their teaching efficacy, thereby increasing their job resources and stimulating the motivational process of JD-R. In light of the aforementioned evidence, the following hypotheses is proposed:

*H3*: Teaching efficacy mediates the relationship between presidents' Transformational Leadership (TL) and faculty Occupational Well-being (OW).

**Figure 1:**
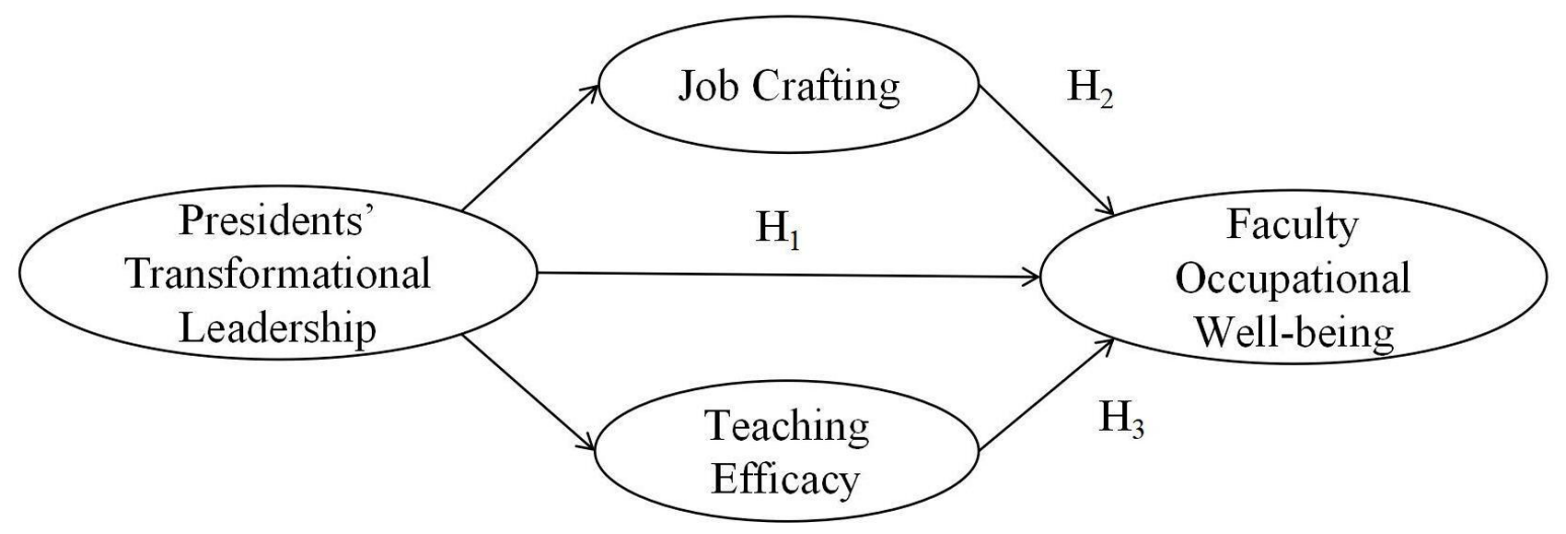
Research hypothesis model.

In light of the aforementioned theoretical elaboration and research hypotheses, we propose a research hypothesis model between presidents’ TL and faculty OW, as illustrated in Figure 1.

## 3 Methods

### 3.1 Participants and procedure

Faculty in China’s higher education institutions typically possess advanced academic qualifications. Their responsibilities encompass teaching, research, administration, and service, often necessitating proficiency in teaching and research, an international perspective, and the capacity to utilize modern information technology. Advancement within this field is frequently contingent upon academic research and teaching excellence. Shandong Province, situated on the eastern coast of China, is one of the country’s most populous provinces. It boasts 167 higher education institutions, encompassing comprehensive universities as well as specialized colleges and universities of science and technology, teacher training, agriculture, and medicine. Higher education faculty in Shandong Province are well represented in terms of type, size, and quality, and are able to exemplify some of the general characteristics of Chinese higher education faculty.

This study used the random sampling technique to conduct a questionnaire survey on faculties in Shandong Province via the Questionnaire Star online platform, which was distributed between May 18 and 22. A total of 600 questionnaires were distributed and 555 were effectively recovered, representing an effective recovery rate of 92.5%. The valid questionnaires were distributed among the following categories of respondents: 275 (49.55%) from undergraduate universities, 185 (33.33%) from teachers at vocational colleges, and 95 (17.11%) from adult higher education institutions. The number of male and female faculty was 253 (45.59%) and 302 (54.41%), respectively. Of the faculty members surveyed, 417 (75.14%) were married, while 138 (24.86%) were unmarried (including divorced and widowed). The distribution of teaching experience among the sampled population revealed the following: 127 (22.88%) of the faculty had 0–5 years of experience, 249 (44.86%) had 6–10 years, 142 (25.59%) had 11–19 years, and 37 (6.67%) had more than 20 years. The distribution of faculty titles is as follows: 223 (40.18%) at the junior level, 280 (50.45%) at the middle level, 31 (5.59%) at the deputy high level, and 21 (3.78%) at the high level. The details are shown in Table 1.

**Table 1:** General demographic characteristics of survey respondents (*n* = 555).

Descriptor	Category	*n*	%	Descriptor	Category	*n*	%
Gender	Male	253	45.59%	Teaching experience	0–5 years	127	22.88%
	Female	302	54.41%		6–10 years	249	44.86%
Marital status	Married	417	75.14%		11–19 years	142	25.59%
	Unmarried (including widowed and divorced)	138	24.86%		≥20 years	37	6.67%
				Title	Junior	223	40.18%
Type of institution	Undergraduate universities	275	49.55%		Middle	280	50.45%
	Vocational college	185	33.33%		Deputy high	31	5.59%
	Adult higher education institutions	95	17.11%		High	21	3.78%

### 3.2 Measures

#### 3.2.1 Occupational well-being

The faculty OW was measured with the 11-item OW scale developed by Zhu (2022). The scale includes three dimensions, namely: Professional Identity (3 items, e.g., I can feel a sense of accomplishment in my job as a faculty member), Organizational Environment Comfort (4 items, e.g., I am satisfied with the teaching and office environment), and Physical and Mental Health Acquisition (4 items, e.g., My job makes me feel stressed, sometimes I have insomnia, anxiety), which is consistent with the concept defined in this study. All items were rated on a 5-point Likert scale (1 = “Strong Disagree,” 5 = “Strong Agreement”), and the higher the score, the greater the OW. Exploratory factor analysis revealed that all items loaded onto three distinct factors, aligning with the scale’s dimensional structure and the theoretical attribution of each item. The Cronbach’s alpha coefficient of the scale in this study was 0.914, with coefficients for the individual dimensions reported at 0.843, 0.895, and 0.874, respectively. These values indicate a high level of internal consistency for both the overall scale and its sub-dimensions. Confirmatory factor analysis found that the structural fit coefficients of the uni-dimensional model (χ^2^/df = 18.182, RMSEA = 0.176, NFI = 0.780, CFI = 0.789, TLI = 0.741) were significantly worse than the multidimensional model (χ^2^/df = 1.292, RMSEA = 0.023, NFI = 0.985, CFI = 0.997, and TLI = 0.996), indicating that the multidimensional scale has good structural validity.

#### 3.2.2 Transformational leadership

The presidents’ TL was measured with the 26-item TL Scale developed by Li and Shi (2005). The scale consists of four dimensions, namely: Visionary Inspiration (6 items, e.g., The president presents an appealing vision of a desirable future), Virtue Exemplar (8 items, e.g., The president prioritize the interests of the organization over their personal interests), Individualized Consideration (6 items, e.g., The president communicates with faculty frequently to gain insight into their work, family, and lives), and Leadership Charisma (6 items, e.g., The president displays an open-minded disposition and evinces a robust commitment to innovation.), which is consistent with the concept defined in this study. All items were rated on a 5-point Likert scale (1 = “Strong Disagree,” 5 = “Strong Agreement”), and the higher the score, the more pronounced the transformational leadership style. Exploratory factor analysis revealed that all items loaded onto four distinct factors, aligning with the scale’s dimensional structure and the theoretical attribution of each item. The Cronbach’s alpha coefficient of the scale in this study was 0.960, with coefficients for the individual dimensions reported at 0.912, 0.936, 0.920, and 0.914, respectively. These values indicate a high level of internal consistency for both the overall scale and its sub-dimensions. Confirmatory factor analysis found that the structural fit coefficients of the uni-dimensional model (χ^2^/df = 9.868, RMSEA = 0.127, NFI = 0.723, CFI = 0.743, TLI = 0.721) were significantly worse than the multidimensional model (χ^2^/df = 1.192, RMSEA = 0.019, NFI = 0.967, CFI = 0.995, and TLI = 0.994), indicating that the multidimensional scale has good structural validity.

#### 3.2.3 Job crafting

The study used the 15-item job crafting scale developed by Tims et al. (2012) and adapted by Zhou (2022). This scale consists of three dimensions: Increasing Structural Job Resources (5 items, e.g., I try to improve my own work ability), Increasing Social Job Resources (5 items, e.g., I hope to be motivated by my superiors), and Increasing Challenging Job Demands (5 items, e.g., I will try to seek for more breakthroughs and challenges in my work), which is consistent with the concept defined in this study. All items were based on a 5-point Likert scale (1 = “Strong Disagree,” 5 = “Strong Agreement”), and the higher the score, the higher the level of job crafting. Exploratory factor analysis revealed that all items loaded onto three distinct factors, aligning with the scale’s dimensional structure and the theoretical attribution of each item. The Cronbach’s alpha coefficient of the scale in this study was 0.954, with coefficients for the individual dimensions reported at 0.912, 0.920, and 0.925, respectively. These values indicate a high level of internal consistency for both the overall scale and its sub-dimensions. Confirmatory factor analysis found that the structural fit coefficients of the uni-dimensional model (χ^2^/df = 13.084, RMSEA = 0.115, NFI = 0.824, CFI = 0.834, TLI = 0.807) were significantly worse than the multidimensional model (χ^2^/df = 1.287, RMSEA = 0.023, NFI = 0.983, CFI = 0.996, and TLI = 0.995), indicating that the multidimensional scale has good structural validity.

#### 3.2.4 Teaching efficacy

The study used the 10-item Teaching Efficacy Scale developed by Tschannen-Moran et al. (1998). This scale consists of two dimensions of General Teaching Efficacy (5 items, e.g., Considering all factors, the influence of faculty on student achievement is minimal) and Personal Teaching Efficacy (5 items, e.g., If I encounter a disruptive or noisy student, I know how to correct them quickly), which is consistent with the concept defined in this study. All items were based on a 5-point Likert scale (1 = “Strong Disagree,” 5 = “Strong Agreement”), and the higher the score, the greater degree of teaching efficacy. Exploratory factor analysis revealed that all items loaded onto two distinct factors, aligning with the scale’s dimensional structure and the theoretical attribution of each item. The Cronbach’s alpha coefficient of the scale in this study was 0.929, with coefficients for the individual dimensions reported at 0.908 and 0.910, respectively. These values indicate a high level of internal consistency for both the overall scale and its sub-dimensions. Confirmatory factor analysis found that the structural fit coefficients of the uni-dimensional model (χ^2^/df = 18.800, RMSEA = 0.127, NFI = 0.828, CFI = 0.835, TLI = 0.788) were significantly worse than the multidimensional model (χ^2^/df = 1.788, RMSEA = 0.038, NFI = 0.984, CFI = 0.993, and TLI = 0.991), indicating that the multidimensional scale has good structural validity.

#### 3.2.5 Control variables

The extant literature indicates that there are differences in faculty OW by gender, with different factors affecting this outcome (Collie and Martin, 2017). Moreover, marital status has demonstrated to impact faculty perceptions of job satisfaction and burnout (Lau et al., 2017). Furthermore, the higher the title, the higher the OW of faculty (Carolan et al., 2017). Accordingly, the present study controlled for gender, marital status, and title as demographic variables. Gender: 1 = male, 2 = female; marital status: 1 = married, 2 = unmarried (including divorced and widowed); and title: 1 = junior, 2 = middle, 3 = deputy high, 4 = high.

## 4 Results

The study used SPSS 27.0 for descriptive and correlational analysis of data and AMOS 26.0 for common method bias test, validation factor analysis, structural equation modeling and hypothesis testing.

### 4.1 Common method biases

Since the data collected in this survey were all results and ratings from faculty self-assessments, the effect of common method bias needed to be excluded. This study used Harman’s single-factor analysis method to test the common method bias. KMO = 0.966 (*p* < 0.001***), indicating the suitability of the data for factor analysis. Following the implementation of exploratory factor analysis on the entirety of the measurement items, it was ascertained that the first factor accounts for 35.31% of variation, which is below 40% (Podsakoff et al., 2003). However, Harman’s single-factor tests cannot fully eliminate concerns about common method bias, we acknowledge this limitation in our study.

### 4.2 Descriptive statistics and correlations

Descriptive statistics and Pearson’s correlation coefficient tests were conducted on the data, and the results are presented in Table 2. Significant correlations (*p* < 0.01) were identified between all four variables: OW was significantly and positively correlated with TL (*r* = 0.654), JC (*r* = 0.512), and TE (*r* = 0.470). TL was significantly and positively correlated with JC (*r* = 0.459) and TE (*r* = 0.394). JC and TE (*r* = 0.505) were significantly positively correlated. The correlations between the variables provide a preliminary basis for subsequent tests of mediating effects.

**Table 2:** Descriptive statistics and correlations.

Variable	*M*	SD	OW	TL	JC	TE
Occupational well-being (OW)	3.303	0.917	1			
Transformational leadership (TL)	3.372	0.869	0.645**	1		
Job crafting (JC)	3.255	0.993	0.512**	0.459**	1	
Teaching efficacy (TE)	3.384	0.959	0.470**	0.394**	0.505**	1

**0.01 level (two-tailed).

### 4.3 Convergent and discriminant validity tests

#### 4.3.1 Convergent validity test

The study used Average Variance Extracted (AVE) values to substantiate the aggregation within variables and the differentiation between variables. In Table 3, the standardized factor loadings β of each measurement dimension exceed 0.7, thereby demonstrating their representativeness of the variables. Moreover, the AVE values of the four variables are all greater than 0.5, and the CRs are all greater than 0.7, indicating satisfactory convergent validity (Fornell and Larcker, 1981).

**Table 3:** Results of convergent validity.

Variable	Measurement variable	*B*	β	S. E.	C. R.	*p*-value	CR	AVE
Occupational well-being	Professional identity	1.000	0.708				0.802	0.576
	Organizational environmental comfort	1.208	0.814	0.073	16.659	<0.001		
	Physical and mental health acquisition	1.042	0.751	0.066	15.679	<0.001		
Transformational leadership	Visionary inspiration	1.000	0.774				0.872	0.631
	Individualized consideration	1.109	0.821	0.056	19.676	<0.001		
	Virtue exemplar	1.027	0.780	0.055	18.645	<0.001		
	Leadership charisma	1.015	0.773	0.055	18.448	<0.001		
Job crafting	Increasing structural job resources	1.000	0.830				0.877	0.705
	Increasing social job resources	1.055	0.853	0.047	22.478	<0.001		
	Increasing challenging job demands	1.072	0.835	0.049	22.002	<0.001		
Teaching efficacy	General teaching efficacy	1.000	0.797				0.795	0.660
	Personal teaching efficacy	1.041	0.828	0.071	14.640	<0.001		

#### 4.3.2 Discriminant validity test

The results of the discriminant validity test for the variables are presented in Table 4. The value on the diagonal represents the square root of the AVE, which indicates the degree of correlation between the internal terms of the variables. The data demonstrate that the external correlations between the variables are all less than the square root of the AVE values of the variables, indicating that the variables are correlated to varying degrees and possess good discriminant validity (Fornell and Larcker, 1981).

**Table 4:** Results of discriminant validity.

Variable	OW	TL	JC	TE
Occupational well-being (OW)	0.759			
Transformational leadership (TL)	0.645**	0.794		
Job crafting (JC)	0.512**	0.459**	0.839	
Teaching efficacy (TE)	0.470**	0.394**	0.505**	0.813
AVE	0.576	0.631	0.705	0.660

**Correlations are significant at the 0.01 level (two-tailed). Diagonal values are square roots of AVE values.

### 4.4 Hypothesis testing and path analysis

**Figure 2:**
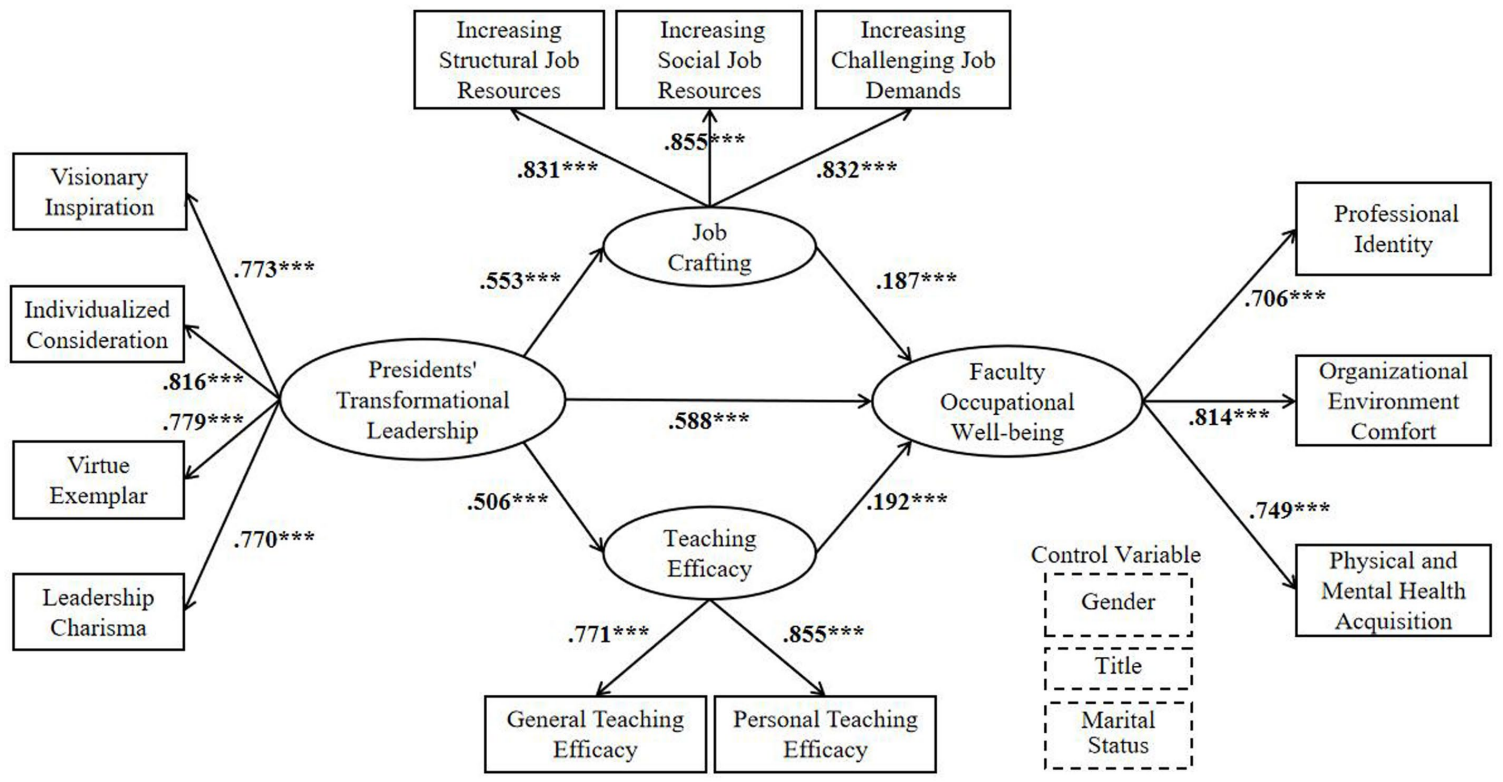
Parallel mediation model of presidents’ TL and faculty OW. The values on the path are standard values. ***p < 0.001.

To test the preceding hypotheses, after controlling for the control variables of gender, title and marital status, a CB-SEM was constructed with presidents’ TL as the independent variable, faculty OW as the dependent variable, and Teaching Efficacy (TE) and Job Crafting (JC) as the mediator variables. OW, TL, JC, and TE are all latent variables. Due to the large number of items in the scale, it can easily lead to large errors and instability, while the item packing method can reduce random errors and improve model quality (Matsunaga, 2008; Wu and Wen, 2011). Therefore, based on the high internal consistency of each dimension in this study, the items were packaged according to theoretical dimensions, and the dimensions were used as observation variables. The CB-SEM is shown in Figure 2. The results of the validated factor analysis on it were: χ^2^/df = 3.193, RMSEA = 0.063, RMSR = 0.064, GFI = 0.942, NFI = 0.925, CFI = 0.947, TLI = 0.935. As Schumacker and Lomax (2004) stipulate, a value of χ^2^/df equal to or less than 5 is deemed acceptable, thus indicating a satisfactory model fit. Therefore, presidents’ TL has a significant direct effect on faculty OW and a significant indirect effect on it through two mediating paths.

To further test the role generated by the mediating variable, the significance of the mediating effect was tested using the bias-corrected percentile Bootstrap method. This method involves repeating the extraction of 5,000 times for the 95% confidence interval. If the confidence interval does not contain 0, it can be concluded that the mediating effect is significant. The results of the test are presented in Table 5.

**Table 5:** Bootstrap mediation effect test.

Results		Paths	Effects	*p*-value	95% CI		Relative mediation effect (%)
					Lower	Upper	
The relationship between variables		TL → JC	0.553	<0.001	0.462	0.639	
		JC → OW	0.187	<0.001	0.074	0.300	
		TL → TE	0.506	<0.001	0.406	0.601	
		TE → OW	0.192	<0.001	0.083	0.304	
The effect of TL → OW	Direct path	TL → OW	0.588	<0.001	0.454	0.709	74.6
	Indirect path 1	TL → JC → OW	0.103	<0.001	0.042	0.166	13.1
	Indirect path 2	TL → TE → OW	0.097	<0.001	0.041	0.158	12.3
	Total effect		0.789	<0.001	0.650	0.860	100

TL, Transformational Leadership; OW, Occupational Well-being; JC, Job Crafting; TE, Teaching Efficacy.

The results demonstrate that the total effect value of the impact of presidents’ TL on faculty OW is 0.789, with a 95% confidence interval of [0.650, 0.860]. Notably, this interval does not contain 0, indicating that the presidents’ TL has a significant positive contributing effect on faculty OW. This finding substantiates the veracity of Hypothesis H1.

Indirect Path 1: TL → JC → OW. The effect value of indirect effect 1 is 0.103, the 95% confidence interval is [0.042, 0.166], and the interval does not contain 0, indicating that job crafting plays a significant mediating role between presidents’ TL and faculty OW, thereby verifying hypothesis H2.

Indirect Path 2: TL → TE → OW. The effect value of indirect path 2 is 0.097, with a 95% confidence interval of [0.041, 0.158], and the interval does not contain 0, indicating that teaching efficacy plays a significant mediating role between presidents’ TL and faculty OW, thereby verifying hypothesis H3.

Direct Path: TL → OW. The effect value of the direct effect was 0.588, the 95% confidence interval was [0.454, 0.709], and the interval did not contain 0, indicating that teaching efficacy and job crafting play a partial mediating role between presidents’ TL and faculty OW.

## 5 Discussion

### 5.1 Presidents’ TL significantly and positively predicts faculty OW

This study found that presidents’ transformational leadership directly and positively predicted faculty occupational well-being (β = 0.588, *p* < 0.001), with 74.6% of the total effect attributed to the direct path. This indicates that presidents’ TL is a substantial determinant of faculty OW. These findings align with those of Liu et al. (2010) and Nielsen and Munir (2009), who observed similar effects of supervisors’ TL on employee well-being in organizational settings. However, unlike these prior studies, which focused on general workplace environments, the present research examines university presidents and faculty in China, thereby extending the scope of TL research into the higher education context.

Some previous studies have suggested that TL may negatively affect well-being—for example, by promoting a vision that encourages self-sacrifice for collective goals (Tourish, 2013) or by motivating employees to take on responsibilities beyond their formal roles, thereby increasing stress (Nielsen and Daniels, 2016). These findings contrast with the present study’s conclusion. In higher education, TL may play a uniquely positive role due to the emotionally intensive nature of academic work. Emotional labor is known to contribute significantly to faculty burnout and reduced OW (Brown et al., 2023). TL encompasses emotionally supportive elements—such as vision, individualized consideration, inspirational motivation, and idealized influence—that can mitigate the adverse effects of emotional labor. By addressing both psychological and emotional needs, TL helps cultivate a positive environment, stimulate intrinsic motivation, and ultimately enhance faculty well-being. Furthermore, within the Chinese cultural context, administrative pressures are a major source of stress for faculty (Yan, 2021), making them especially responsive to the positive influence of transformational leadership.

### 5.2 Job crafting partially mediates the predictive role of presidents’ TL and faculty OW

The present study found that job crafting partially mediated the predictive effect of presidents’ TL on faculty OW (β = 0.103, *p* < 0.001), with a mediating effect share of 13.1%. This finding suggests a potential mechanism by which presidents’ TL predicts faculty OW through the mediating role of job crafting. Previous study has found that TL promotes certain job crafting behaviors (Naeem et al., 2020), and job crafting improves work engagement and performance (Moreira et al., 2022). This study bridges the gap in research on the relationship between job crafting and OW, confirms the predictive effect of job crafting on OW, and explore the relationship between presidents’ TL, job crafting, and faculty OW from a more comprehensive perspective. In conclusion, presidents’ TL facilitates faculty job crafting by optimizing their job demands and resources, expanding their job autonomy (Hetland et al., 2018), which enhances the perception of positive factors at work, stimulates the “Motivational Path” in JD-R theory, and ultimately enhance faculty OW. This finding adds a new perspective to our understanding of how TL affects OW and expands the application of JD-R theory.

### 5.3 Teaching efficacy partially mediates the predictive role of presidents’ TL and faculty OW

This study indicated that the teaching efficacy of faculty members exerted a partial mediating influence on the predictive effect of presidents’ TL on faculty OW (β = 0.097, *p* < 0.001), accounting for a mediating effect share of 12.3%. This finding reveals an additional potential mechanism through which presidents’ TL predicts faculty OW via the mediating role of teaching efficacy. It can be proposed that an increase in teaching efficacy will result in an increase in faculty OW. This finding validates the contribution of TL to teaching efficacy in existing research studies (Bellibas and Liu, 2017) and the relationship between teaching efficacy and well-being (Reppa et al., 2023). The mediation path introduced in this study highlights the important role of teaching efficacy in the relationship between presidents’ TL and faculty OW. In conclusion, presidents’ TL, through intellectual and motivational stimulation and other behaviors, enables faculty to make accurate assessments of their own abilities, and improves their beliefs in facing the difficulties and challenges of teaching in the new era, which improves teaching efficacy. The improvement of teaching efficacy can help faculty obtain more job resources, stimulate the “Motivational Path” in JD-R theory, and ultimately enhance faculty OW. The finding helps us to understand the internal action mechanism of the relationship more deeply, and the mechanism analysis based on the JD-R theory enriches the scope of application of the theory.

## 6 Suggestions

This study investigates the mechanism through which university presidents’ transformational leadership influences faculty occupational well-being through the lens of the Job Demands-Resources theory. Based on the findings, several recommendations are proposed to enhance faculty OW. First, it is crucial to implement a change management strategy aimed at improving presidents’ TL. The study demonstrates that TL has a significant positive impact on faculty OW, making it vital to reinforce TL practices. Research indicates that TL skills can be developed through education, training, and coaching (Schmitt et al., 2016). As such, incorporating TL-related courses into president training programs would help leaders understand the concept and characteristics of TL, promoting innovative and supportive practices. Presidents should also focus on developing and effectively communicating a compelling vision for the institution’s future, as this can inspire faculty and align the institution’s goals with those of faculty (Steinmann et al., 2018). Furthermore, in line with Noddings (1995), presidents should prioritize individualized care for faculty. They can engage with faculty through discussions, interviews, and other personal interactions, offering material, emotional, and psychological support to foster a sense of connection and well-being.

The study also highlights the importance of optimizing the balance between job demands and resources, facilitating job crafting for faculty. Given that job crafting significantly predicts faculty OW, it is necessary to create a work environment where job demands and resources are aligned with faculty needs. This can be achieved by designing stimulating and meaningful tasks, reducing unnecessary bureaucratic requirements, and introducing more challenging roles that motivate faculty (Moreira et al., 2022). Job crafting interventions should be incorporated into faculty development programs, with tailored training and personalized interventions that help faculty actively engage in job redesign. Additionally, faculty should take initiative in managing their roles by developing job crafting plans and reflecting on their effectiveness. Clear understanding of their job demands and resources will empower faculty to craft their work in a way that enhances motivation and well-being.

Finally, enhancing faculty teaching efficacy is essential for improving OW. The study reveals that teaching efficacy is a significant predictor of faculty well-being, making it crucial for higher education management to foster a supportive environment that nurtures teaching efficacy. University leaders should adopt individualized and visionary leadership approaches, offering tailored guidance and professional development opportunities for faculty. Increasing faculty autonomy in areas like curriculum design and teaching decisions will further strengthen their sense of ownership and commitment, thereby improving teaching efficacy. Additionally, while external support is important, faculty themselves need to cultivate internal motivation for teaching efficacy. By engaging in continuous professional development, learning new teaching techniques, and enhancing their classroom management skills, faculty can boost their confidence and effectiveness, which in turn improves their teaching efficacy and overall well-being.

## 7 Conclusion, limitations and future directions

This study was grounded in the JD-R theory and developed a parallel mediation model. The independent variable was presidents’ Transformational Leadership (TL), while the mediators were Job Crafting and Teaching Efficacy, with faculty Occupational Well-being serving as the outcome variable. The key findings are as follows: presidents’ transformational leadership has a significant and positive effect on faculty occupational well-being; teaching efficacy partially mediates the relationship between presidents’ transformational leadership and faculty occupational well-being; and job crafting also partially mediates the relationship between presidents’ transformational leadership and faculty occupational well-being.

However, the present study has several limitations that warrant further exploration in future research. First, while the mediating effects are significant, the combined effects of the two mediating variables account for only 25.4% of the total variance. This suggests that, beyond job crafting and teaching efficacy, other mediating factors could be influencing faculty well-being, which were not addressed in this study. Future research could explore additional mediators and moderating variables (such as organizational culture or psychological safety) to broaden the scope of investigation. Second, this study relied on self-reported data, which may introduce common method biases, despite performing Harman’s single-factor test. Future research could use multi-source data to mitigate these biases. Additionally, the cross-sectional design of this study limits causal interpretations. Longitudinal studies could provide a more comprehensive understanding of the changes over time or under varying conditions, offering clearer insights into causal relationships.

## Data Availability

The raw data supporting the conclusions of this article will be made available by the authors, without undue reservation.
